# DTI Analysis in Patients with Primary Open-Angle Glaucoma: Impact of Registration on Voxel-Wise Statistics

**DOI:** 10.1371/journal.pone.0099344

**Published:** 2014-06-05

**Authors:** Manuel A. Schmidt, Angelika Mennecke, Georg Michelson, Arnd Doerfler, Tobias Engelhorn

**Affiliations:** 1 Department of Neuroradiology, Friedrich-Alexander-University Erlangen-Nuremberg, Erlangen, Germany; 2 Department of Ophthalmology, Friedrich-Alexander-University Erlangen-Nuremberg, Erlangen, Germany; Institute of Psychology, Chinese Academy of Sciences, China

## Abstract

**Background and Purpose:**

Tract-based spatial statistics (TBSS) has been used to assess the integrity of the visual pathway in glaucoma patients. TBSS uses the subjects’ FA data to create a mean FA skeleton of white matter tracts before running voxel-wise cross-subject statistics. We compared four different approaches of registration of FA maps to create the skeleton and evaluated alignment and subsequently the impact of the chosen registration on voxel-wise statistics.

**Material and Methods:**

Our study comprised 69 subjects, i.e. 46 patients with primary open angle glaucoma (POAG) and a healthy, age-matched control group of 23 subjects. Mean FA skeletons were created using the following registration approaches: registration to a standard template (T), registration to the group mean (GM), registration to a group-wise atlas (GW) and registration to the most typical subject (N). Subsequently, maps of standard deviation of the 4D images were created to assess the alignment. Voxel-wise statistics for each registration approach were performed.

**Results:**

We found distinct differences in voxel-wise statistics depending on the chosen registration approach. Best alignment results were achieved by registration to a study specific template, i.e. to the group mean (GM) or to a group-wise atlas (GW). Overall alignment did not differ between these two approaches. However, voxel-wise statistics showed clusters of significantly decreased FA values in the T and GM approach, which were not significant after GW registration. These voxels of significantly decreased FA values after T and GM registration did not represent white matter tracts and correlated with higher standard deviation in FA maps across subjects, thus implying registration errors, especially in the optic radiation.

**Conclusion:**

Registration to a study-specific template, i.e. to the group mean or a group-wise atlas seems to be the method of choice in TBSS-analysis of glaucoma patients as it shows better alignment of the optic radiation and helps to rule out registration errors due to misalignment.

## Introduction

Glaucoma is a complex neurodegenerative disease that is characterized by neuronal degeneration of the whole visual pathway [1]. There has been some research on examining the visual pathway with different methods. Voxel-based morphometry analysis for instance showed significant volume reduction of the structures of the visual pathway [2], [3]. Furthermore, DTI data sets and FA values of glaucoma patients have been used as a surrogate parameter for the integrity of the visual pathway (3^rd^ and 4^th^ neuron). Engelhorn et al. specified certain regions of interest (ROIs) and found a decrease of FA values in the optic nerve and in the optic radiation, respectively, significantly correlating with ophthalmological examinations concerning disease severity [4]. Lately, voxel-wise statistical analysis of FA data of glaucoma patients has been conducted using tract-based spatial statistics (TBSS) [5]–[7]. TBSS uses the subjects’ FA data to create a mean FA skeleton of white matter tracts before running voxel-wise cross-subject statistics [8], [9]. Particularly in case of analyzing FA data of glaucoma patients with TBSS, correct alignment of FA maps is most important referring to the anatomy of the optic pathway. Hence, we compared four different approaches of registration of FA maps and evaluated alignment and the impact of the chosen registration on voxel-wise statistics.

## Materials and Methods

### Subjects

We included a total of 69 subjects in our study: 46 severely affected patients with primary open angle glaucoma (POAG, intraocular pressure of both eyes prior to treatment ≥22 mmHg; mean age = 63.75 years +/−10.79) and 23 healthy, age-matched control individuals (CONT, mean age = 59.54 years +/−14.06). The individuals of the CONT group underwent full ophthalmological examination to exclude an intraocular pressure ≥22 mmHg, optic nerve head atrophy and visual disturbances. Patients’ characteristics are summarized in table 1. Informed consent was obtained from all subjects. The Clinical Investigation Ethics Committee of the University of Erlangen-Nuremberg approved the study protocol and the research was conducted in accordance with the Declaration of Helsinki.

**Table 1:** Clinical characteristics of POAG patients, given as mean +/− standard deviation.

	right eye	left eye
Age	63.75+/−10.79	
IOP, treated (<22 mmHg)	16.9+/−4.2	17.3+/−4.3
HRT disc area (1.69–2.82 mm^2^)	2.326+/−0.596	2.353+/−0.643
HRT cup area (0.26–1.27 mm^2^)	1.051+/−0.512	1.108+/−0.611
HRT rim area (1.2–1.78 mm^2^)	1.276+/−0.511	1.246+/−0.613
RNFL thickness (0.18–0.31 mm)	0.194+/−0.062	0.194+/−0.124
FDT duration (≤50 s)	66.7+/−34.8	72.0+/−33.0

Reference value in brackets. IOP = intraocular pressure; HRT = Heidelberg Retina Tomograph; RNFL = retina fiber layer thickness; FDT = frequency doubling test.

### Imaging Protocol

We used a 3T high-field scanner (Magnetom Tim Trio, Siemens Healthcare AG, Erlangen, Germany) with a gradient field strength up to 45 mT/m (72 mT/m effective). DTI was performed in the axial plane with 4 mm slice thickness using a single-shot, spin echo, echo planar imaging (EPI) diffusion tensor sequence (TR = 3400 ms, TE = 93 ms, FoV = 230×230 mm^2^, acquisition matrix size = 256×256 reconstructed to 512×512, number of signal averages = 7, partial Fourier acquisition = 60%). Diffusion weighting was carried out with a maximal b-factor of 1000 s/mm2 along 15 icosahedral directions complemented by one scan with b = 0.

### Image Analysis

DICOM images were converted to NIfTI files (Neuroimaging Informatics Technology Initiative) using dcm2nii from the MRIcron – package (http://www.mccauslandcenter.sc.edu/micro/mricron/dcm2nii.html). The resulting images were then preprocessed for TBSS analysis, i.e. corrected for eddy currents with eddy_correct and brain extracted with bet2 [10]. We chose a bet2 - threshold of 0.2 to remove all non-brain tissue because we focused on the 4^th^ neuron of the visual pathway in our analysis. Next a diffusion tensor model was fit at each voxel to extract the FA maps using DTIFIT. All of these preprocessing steps were carried out using FMRIB Diffusion Toolbox (FDT) which is part of FSL (http://fsl.fmrib.ox.ac.uk/fsl) [11]. We used the JHU White-Matter Tractography Atlas for annotation [12], [13].

For TBSS analysis, images were reviewed carefully and faulty images with quality issues were sorted out. We used four different registration approaches, to an atlas or to a single image as follows:

Registration to an atlas:

- Registration to a standard template (T): All FA images were nonlinearly registered to the FMRIB58_FA standard template in the MNI152 standard space. FMRIB58_FA is an average of 58 FA images of healthy male and female subjects 20 to 50 years of age (http://fsl-fmrib.ox.ac.uk/fsl/fslwiki/FMRIB58_FA).
- Registration to the group mean (GM): As previously, the images were nonlinearly registered to the FMRIB58_FA standard template. An average mean FA map was created and the original FA images were nonlinearly registered to this group mean. This approach has been used before in TBSS analysis of patients with Alzheimer’s disease who had ventricular enlargement due to atrophy [14].
- Registration to a group-wise atlas (GW): All of the above are standard options of the TBSS script or a combination of these. Based on the approach of Keihaninejad et al., we created a group-wise atlas as target for the registration. Therefore, a random image of the group was selected firstly, to which the other FA images were rigistered rigidly. That step was followed by four affine and ten nonlinear registrations to the respective antecedent mean FA image. Every non linear registration was followed by an affine registration to FMRIB58_FA [15].

Registration to a single image:

- Registration to the most typical subject (N): Every FA image was aligned to every other one; the most typical subject was identified by the minimum of warping which was required for the other images to align to it. The identified most representative image of the group was then aligned into the MNI152 standard space and served as the target for registration [8].

To assess alignment, we calculated the residual standard deviation of the warped FA images for every registration approach voxel by voxel using fslmaths, which is also part of FSL. We estimated the probability density function of the non-zero voxels of the respective standard variation image using the Matlab statistics toolbox (MATLAB and Statistics Toolbox Release R2011b, The MathWorks, Inc., Natick, Massachusetts, United States).

Statistical analysis was carried out through an unpaired t-test using randomise (part of FSL) with two contrasts: contrast 1 POAG>CONT and contrast 2 POAG<CONT, permutations = 5000, corrected for multiple comparisons over space by controlling the family-wise error rate, using threshold-free cluster enhancement at p<0.05 [16].

## Results

For contrast 1 no differences between the registration methods could be revealed: POAG patients showed no voxels of significantly increased FA values compared to healthy controls in all registrations.

**Figure 1:**
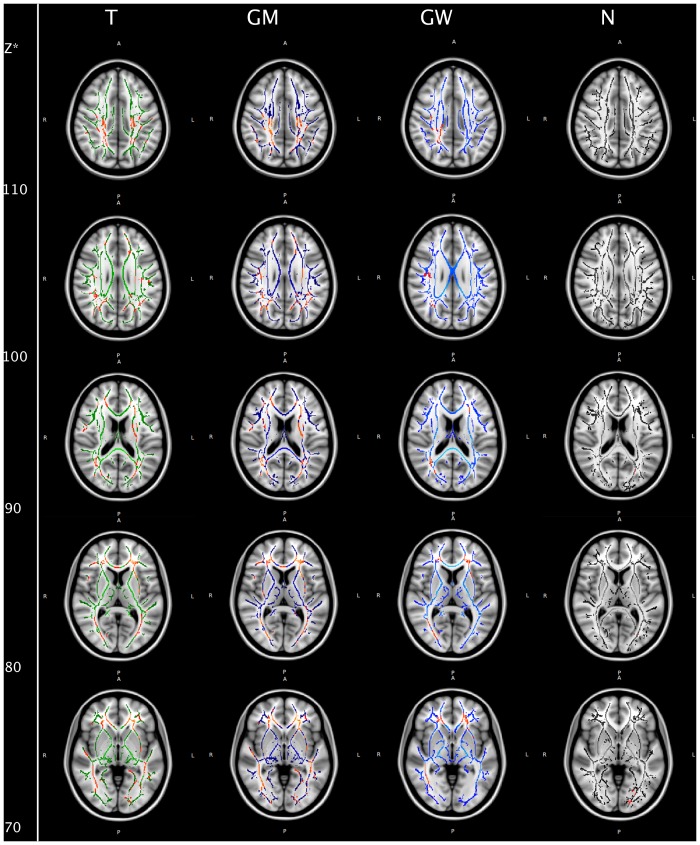
Voxel-wise statistics. Mean FA skeleton for each registration approach: green (T, standard template), blue (GM, group mean), light blue (GW, group-wise atlas) and black (N, most representative subject) overlaid onto the MNI152 standard template for anatomical orientation. Clusters of voxels of significantly decreased FA values of POAG patients compared to controls are marked red (p<0.05, corrected for multiple comparisons). *Coordinate in MNI_152 space.

**Figure 2:**
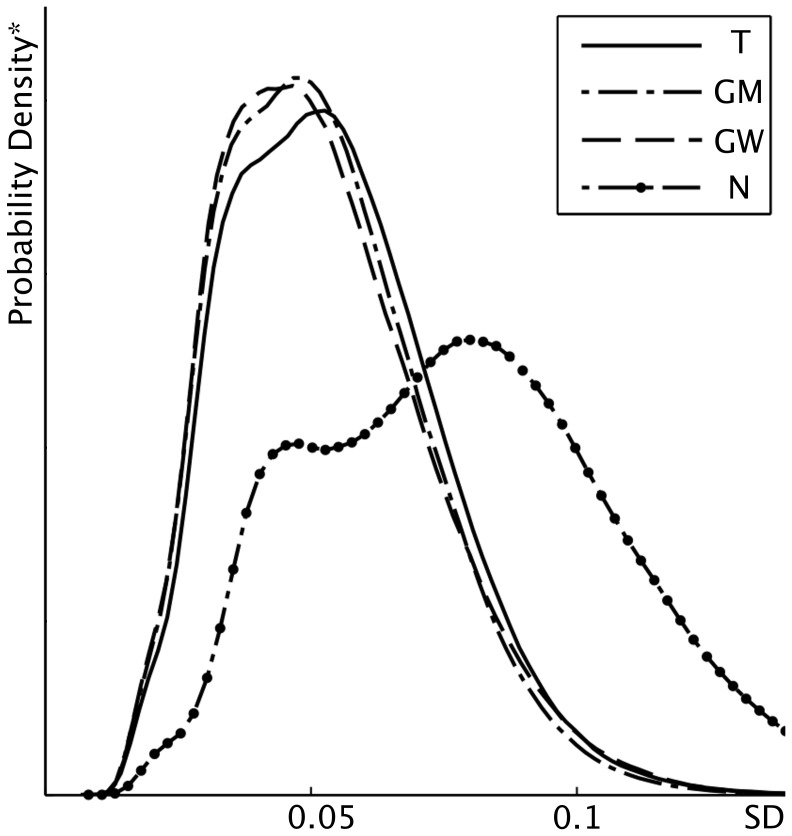
Density plot for standard deviation (SD). Plot of the estimated probability density function of the residual standard deviation of the non-zero voxels of all aligned FA maps for the different registration approaches. *Regarding to the definition of the probability density function, the y-range is calculated as to norm the integral of the function to one.

**Figure 3:**
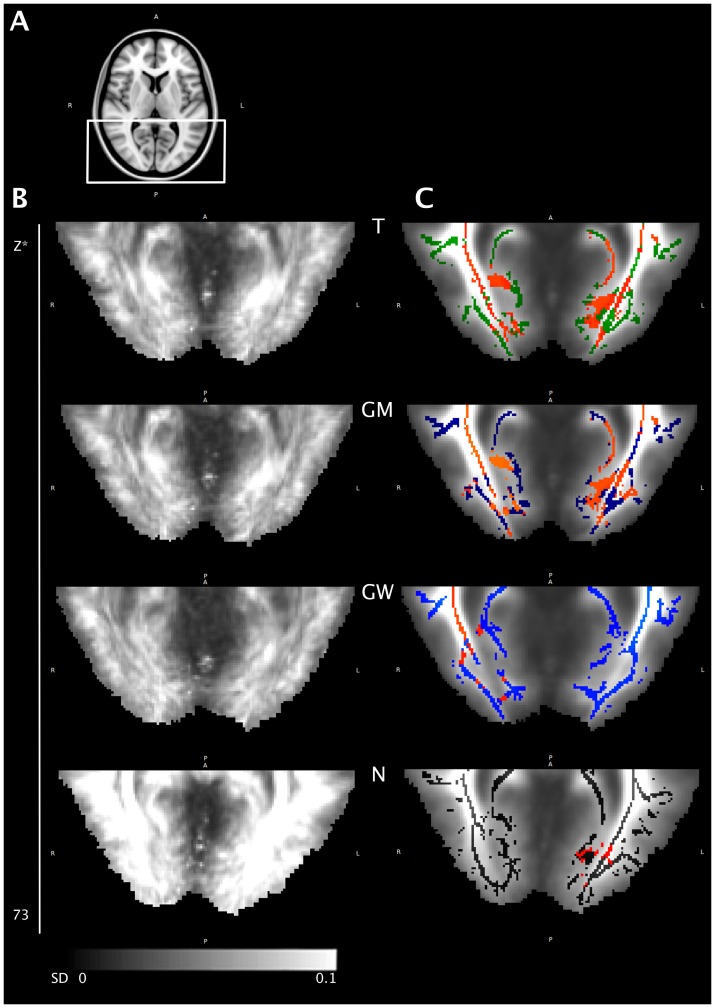
Voxel-wise statistics, axial ROI. (A) Axial ROI. (B) Standard deviation map of all aligned FA maps for each registration approach. (C) Mean FA skeleton for each registration approach: green (T, standard template), blue (GM, group mean), light blue (GW, group-wise atlas) and black (N, most representative subject) overlaid onto the mean FA image of the respective registration approach. Clusters of voxels of significantly decreased FA values of POAG patients compared to controls are marked red (p<0.05, corrected for multiple comparisons). *Coordinate in MNI_152 space.

For contrast 2 we found multiple white matter tracts with clusters of voxels of decreased FA values in glaucoma patients compared to CONT, summarized in table 2. These clusters differ distinctly depending on the registration method that had been applied (Figure 1, Table 2). Standard deviation (SD) of the aligned FA maps of all subjects was lowest for the GW and GM registration, followed by the T and N approach with no noticeable difference between GM and GW in overall alignment (Figure 2). For the optic radiation, lower SD could be detected for the GM and GW approach. Voxels of significantly decreased FA values in the T and GM approach, that were found not to be significant in the GW approach, do either not correspond to white matter tracts in the respective mean FA image or fail to reach statistical significance (Figure 3).

**Table 2:** White matter tracts with clusters of significantly decreased FA values in POAG patients compared to controls (p<0.05, corrected for multiple comparisons) for different registration approaches.

	Z*	T	GM	GW	N
right corticospinal tract	120	x	x	x	x
left corticospinal tract	120	x	x		
right superior longitudinal fasciculus	110	x	x	x	
left superior longitudinal fasciculus	110	x	x		
forceps minor right	100	x	x	x	
forceps minor left	100	x	x	x	
right inferior longitudinal fasciculus	100	x	x	x	
left inferior longitudinal fasciculus	100	x	x	x	x
right fronto-occipital fasciculus	100	x	x	x	
left fronto-occipital fasciculus	100	x	x	x	x
forceps major right	100	x	x	x	
forceps major left	100	x	x		x
right posterior thalamic radiation	90	x	x	x	
left posterior thalamic radiation	90	x	x		
right internal capsule	90				
left internal capsule	90	x	x		
right anterior corona radiata	80	x	x	x	
left anterior corona radiata	80	x	x	x	
right capsula externa	80		x		
left capsula externa	80	x	x	x	
genu of corpus callosum	80	x	x		
splenium of corpus callosum	80		x		
right cingulum	70				
left cingulum	70	x	x		
right uncinate fasciculus	60		x		
left uncinate fasciculus	60	x	x		

T = standard template; GM = group mean; GW = group-wise atlas and N = most representative subject. *Coordinate in MNI_152 space.

## Discussion

Our results reveal decreased FA values in glaucoma patients compared to controls not only in the optic radiation (4^th^ neuron) but also in white matter tracts not directly related to the visual pathway. Furthermore, we demonstrated that tract-based voxel-wise statistics in glaucoma patients strongly depend on the registration method that has been applied in the TBSS pipeline and that best alignment can be achieved when choosing a study specific template for registration.

In glaucoma patients a decrease of FA values, as correlate of neurodegeneration, can be found in various structures, not only in the visual pathway. Reduced gray matter in VBM analysis of POAG patients in the calcarine fissure, the postcentral gyrus, the superior frontal gyrus and the inferior frontal gyrus has been reported [17]. That corresponds to our results of decreased FA values in the important association fibers, as transneuronal degeneration seems to play an important role in the pathogenesis of glaucoma [17], [18]. Decreased FA values in projection fibers such as the internal capsule have been described before and may also be related to the pathogenesis of glaucoma [7]. Our results are in line with these reports, however, the significance of these alterations in white matter tracts not directly linked to the visual pathway still remains to be clarified concerning the correlation of these finding with clinical parameters of glaucoma patients. Moreover, further studies are necessary to elucidate if there are detectable differences between different types of glaucoma using TBSS.

At least for FA changes in the optic radiation of POAG patients, correlation to indices of glaucoma severity has been described [19].

Focusing on the 4^th^ neuron of the visual pathway, we found decreased FA values in the optic radiation in POAG patients, which is also in line with previous reports where TBSS [5], [6] or statistical parametric mapping (SPM) [20] were used for DTI analysis. Hereby, all previous TBSS studies of glaucoma patients are based on registration to a standard template. Hence, our purpose was to evaluate the impact of different registration approaches on voxel-wise statistics. We could demonstrate that the statistics strongly depend on the chosen registration method, particularly in the optic radiation. When comparing the overall standard deviation of the warped 4D images of the four registration approaches (Figure 2) it is clearly evident that registration to a study specific template, i.e. to the group mean or a group-wise atlas, performed best, suggesting better alignment when using one of these registration methods. One reason for the differing results in the statistics might thus be misalignment, with subsequent false positive results. This is particularly obvious in the occipital periventricular white matter, where voxels of significantly decreased FA values in the T and GM approach do not correspond to white matter tracts in the respective mean FA image but to the grey and white matter junction. Moreover, GW registration was solely able to generate a symmetric skeleton in that ROI, possibly hinting at correct alignment.

For the left optic radiation, the left inferior longitudinal and the left inferior fronto-occipital fasciculus (Figure 3) misalignment does not suitably explain the absence of significantly decreased FA values in those tracts. Those voxels might be false negative in the GW approach rather than false positive in the GM approach. There is a strong trend, as clusters of decreased FA values in that ROI scarcely failed to reach statistical significance after GW registration. Thus, another reason for differing results in voxel-wise statistics, particularly in this ROI, might be partial volume effects regarding slice thickness in the initial DTI datasets that account for differing FA values in those voxels not reaching statistical significance.

One obstacle for good alignment results is the variety of ventricular size and the extent of brain atrophy. This is particularly important when analyzing glaucoma patients, as the optic radiation is running in immediate spatial neighborhood to the ventricles. While TBSS style analysis per se tries to overcome this problem through projection of FA values onto a mean FA skeleton we show that there are, nevertheless, distinct differences in voxel-wise statistics depending on the chosen registration approach.

It has been proposed that registration to a real FA image rather than to an averaged (atlas) image delivers better alignment results [8]. Our results are contradictory as registration to the most representative subject of the study group showed the highest SD with only few voxels of significantly different FA values, probably representing registration errors due to misalignment. In contrast, registration to a study specific atlas, the group average or a group-wise atlas, showed far better alignment results then registration to a standard template. TBSS seems not to be able to sufficiently overcome rough misalignment due to ventricular enlargement and brain atrophy in the study group when registering the study group to an unmatched template, such as FMRIB58_FA.

Registration to a study specific template helps to rule out registration errors in the optic radiation. This might be especially important for further analysis of various glaucoma types and may help to monitor treatment impacts. For further TBSS analysis of the visual pathway in glaucoma patients, registration to a study specific template through averaging or a group-wise atlas seems to be the method of choice.

## Conclusion

Registration to a study specific template should be conducted when performing tract-based statistical analysis of glaucoma patients as it shows better alignment results than registration to a standard template and helps to rule out registration errors in voxel-wise statistics. This might be of special interest for analysis of different types of glaucoma as well as to monitor treatment impacts.
